# Agri-Food Side-Stream Inclusions in the Diet of *Alphitobius diaperinus* Part 1: Impact on Larvae Growth Performance Parameters

**DOI:** 10.3390/insects11020079

**Published:** 2020-01-23

**Authors:** Natasja Gianotten, Lise Soetemans, Leen Bastiaens

**Affiliations:** 1 Protifarm, Harderwijkerweg 141B, 3852 AB Ermelo, The Netherlands; n.gianotten@Protifarm.com; 2 Flemish Institute for Technological Research (VITO), Boeretang 200, 2400 Mol, Belgium; lise.soetemans@vito.be

**Keywords:** agri-food side-streams, insect diets, insect growth, lesser mealworm

## Abstract

Insects are attracting increased attention in western countries as a protein source for feed and food industries. Currently, insect farmers use high-quality (cereal-based) diets. Part of the ingredients in these diets can also be used directly in food applications. To avoid competition and improve the sustainable and economical aspect of insect rearing, a search for alternative insect diets is ongoing. Side-streams from the agri-food sector offer potential. The lesser mealworm (*Alphitobius diaperinus*) is an insect that is commercially reared on large scale for food application. The current paper reports on six agri-food side-streams that were included in the diet of the lesser mealworm. The impact of 29 diets (single side-streams or mixtures) on the larvae growth was evaluated by monitoring the larval yield, efficiency of conversion of ingested feed, and larval weight. The larvae were able to grow on all diets, but differences in growth were observed. Two side-streams, wheat middlings and rapeseed meal, were proven to support good larval performance when used as a single ingredient. A combination of these two with brewery grains as moisture source provided (1) the best larval growth and (2) the most economically profitable diet. In conclusion, this study illustrates successful rearing of the lesser mealworm on side-stream-based diets.

## 1. Introduction

During the last decades, a growing trend towards recycling and minimizing waste is noticeable. The European Commission stimulates this trend by encouraging the consideration of ‘waste’ (comprising agri-food side-streams) as a new resource via re-use and recycling [1]. Side-streams that are not usable for food applications can be converted, by insects, to a new raw material for food and/or feed applications. According to DeFoliart “Practically every substance of organic origin, including cellulose, is fed upon by one or more species of insects, so it is only a matter of time before successful recycling systems will be developed” [2]. Because of the lower feed-to-edible biomass conversion ratio of insects compared to other livestock animals (about 4.7 for the yellow mealworm (YM) and lesser mealworm (LM), reared on side-streams [3,4] versus 24 for beef [5]), insects are a promising protein source for the future [5]. Up until now, the larvae of the black soldier fly, the common housefly, and larvae of mealworm species from the Tenebrionidae family are seen as promising insects for industrial rearing and were proven to be very efficient in reducing the volume of organic remains [5,6]. The larvae of mealworm species from the Tenebrionidae family, such as the yellow mealworm (YM, *Tenebrio molitor*), the lesser mealworm (LM, *Alphitobius diaperinus*), and the superworm (*Zophobas morio*), are reared as feed for reptile, fish, and avian pets. *T. molitor* and *A. diaperinus* are also considered particularly fit for human consumption and are offered as human food in specialized shops [5].

Most insect rearing companies use commercial feed or cereal-based raw material because species-specific diets are not yet commercially available for all insect species [7]. Mealworms are usually reared on mixed grain diets [3,8]. On one hand, the grain ingredients used in these mixed grain diets can be considered beneficial because these are also directly used in food production and thus do not need extra precautions considering food/feed safety legislation. On the other hand, competition from the feed and food industry for grain products will become a problem. The substitution of grain products with side-streams can help to make insect farming more profitable and sustainable [9,10]. For example, Van Zanten et al. (2015) reported environmental impact data on the use of side-streams for growing pigs. For instance, the environmental impact of wheat middlings is lower compared to wheat in terms of global warming potential (237 g CO_2_-eq/kg versus 367 g CO_2_-eq/kg), energy use (2.0 MJ/kg versus 2.7 MJ/kg), as well as land use (0.6 m^2^/kg versus 1.1 m^2^/kg). Preferably, the selected side-streams would be underspent and not suitable as a diet component for other animals to avoid competition with other feed applications.

The option to use side-streams for mealworm rearing has been reported before. Oonicnx et al. (2015) tested, among others, a mixture of freeze-dried side streams (spent grains, beer yeast, and cookie remains) that supported good growth of the YM [4]. Compared to a control diet, the mixture was able to support similar survival rate of YM (84% for the control and 79% for the side-stream mixture) and decreased the development time (116 days versus 145 days). Van Broekhoven et al. (2015) reported similar findings when rearing the LM on a similar diet [3]. A mixture of maize DDGS (Distillers Dried Grains and Solubles), beer yeast, bread remains, and potato peels resulted in a higher larval survival (95%) compared to the control diet (79%–82%) with similar development time (44 days versus 42–66 days) and the same pupal weight (0.022 g). By replacing the potato peels by spent grains, the development time was even shorter (38 days), whereas the survival and pupal weight were the same as the control. Ramos-Elorduy et al. (2009) successfully even reared the YM on a mixture of freeze-dried cereals, fruit, and vegetables [11] with a larval weight varying between 0.07 and 0.11 g/larva. These studies indicate that the mealworm can be reared on a broad range of diet compositions. However, all previous studies reported on freeze-dried food side-streams or were oven dried at 90 °C [12]. When envisioning rearing on a large scale, this pre-treatment is not economically feasible. 

The current study focusses on the growth of the LM reared on six non-freeze-dried side-streams that can be used ‘as such’ in order to identify cheaper, and possibly more sustainable diets [13,14], suitable for mass rearing. Side-streams originating from grain products (corn DDGS, rice bran, wheat middlings, corn gluten feed, and brewery grains), instead of the grain products itself, were considered, as well as rapeseed meal, a side-stream originating from the oil industry.

## 2. Materials and Methods

**Insects and housing**: Larvae of the lesser mealworm (LM, *Alphitobius diaperinus*) were reared on a semi-industrial scale (about 45,000 larvae per tray) during a growth period of 28 days. The same strain was used over a period of 1 year, which originated from the Kreca/Protifarm company and that was stable for 30 years. Larvae were housed in open, stacked, plastic trays (40 × 60 × 12 cm, shaded from light), which were kept in a temperature and humidity-controlled room, with standard a temperature ranging between 28 and 32 °C and humidity above 60%.

**Diet ingredients**: During selection of the side-streams, a dry matter (DM) content requirement of at least 80% was taken into account (Table 1). Side-streams originated from feed-grade materials or were bought on the feed-market (viz., the brewery grains). Grated carrots (bought in a local store, 9% DM, 6% protein on DM basis, 1.6% lipids on DM basis [4]) or brewery grains (BG, 25.7% DM, 25.8% protein, 60 €/ton) were provided as a moisture source.

All side-streams were finely ground (Hammermill, 1 mm sieve) to improve the feed intake by the LM. Pure side-streams, as well as mixtures, were tested (see Table 2). Moisture sources were provided according to a feeding schedule that secured optimal moisture according to the growth phase of the larvae. 

**Rearing and harvesting:** After hatching, a weighed amount of first instar larvae (the starting weight of first instar larvae was kept constant per tray throughout all experiments) were reared immediately on the chosen diet. Larvae were provided with the diets ad libitum (according to the feeding schedule that was designed for optimal rearing that was based on years of experience) and checked every day for shortages or abundance on the basis of the color differences between the feed and LM manure. When the larvae grew at a different rate, the feeding deviated from the schedule. After 14 days, a representative sample of the growth medium along with larvae (about 150 to 300 individuals, being 0.5% of the larvae) was taken out of the tray. Before subsampling, the content of the tray was mixed with a spoon and a subsample (substrate + larvae) was taken out carefully. The larvae were separated from the substrate, and subsequently weighed and counted twice for calculating an average larval weight (weight per larvae) at day 14. Further rearing of the remaining larvae till commercially ‘mature’ larvae lasted a total of 28 days. The larvae were subsequently separated from the residue by sieving. After sieving, the larval yield was measured and a representative subsample of clean larvae was weighed and counted twice to calculate the average weight per larvae on day 28.

**Experimental set-up:**Table 2 summarizes the inclusion percentages of the side-streams in all diets tested and the type of moisture source. Firstly, pure side-streams (diet A until diet I) were tested with two different moisture suppliers. Carrots are often used in the experimental phase because they only provide moisture and almost no nutrients, making them a good source to test the ability of the side-stream to insure successful rearing. However, on an industrial level, carrots need to be replaced because they are not easily scalable to larger rearing facilities nor economically favorable. In this study, BG was chosen as a second moisture source. This is a side-stream that is naturally wet (±75% moisture) but also provides nutrients. Secondly, side-stream mixtures (diet J until diet AC) were made with diet F (100% wheat middling with BG as moisture) as a fundamental basic diet, and small percentages were replaced by another side-stream to generate a more diverse diet. In every trial, the reference diet was included. The evaluation of the mixed diets was based on improvement or decrease in larval performance compared to the reference diet (in the same trial) without the inclusions (diet F).

**Calculations and statistics:** The performance of the larval growth was monitored by determining (1) the larval yield (grams of fresh larvae per tray determined at the end of the rearing period after separation of the residue), (2) individual larval weight at day 14 and day 28 (milligrams per larvae), (3) the bioconversion efficiency (BE), and (4) the total amount of feed given until the end of the trial (weight of feed (kg)). The BE was used to evaluate the feed conversion efficiency and was calculated according to Bosch et al. (2019) [16], as shown in Equation (1). The starting mass of the larvae was presumed zero because the first instar larvae were directly put on the tested feed and the mass was negligible. The DM of LM larvae (35%) and the side-streams were based on the findings of Yi et al. (2013) or the CVB table (Federatie Nederlandse Diervoederketen, 2018), respectively.

(1) $$BE=\frac{g\;dry\;larvae\;after\;rearing-g\;dry\;larvae\;begin}{weight\;of\;feed\;provided\;\left(DM\right)}\times 100.$$

Wheat middlings with BG as a moisture source (diet F) was selected as the reference diet and was included in every test set. Preliminary tests indicated that this diet had the best larval performance of all side-streams and had, in comparison to industrial rearing, a reasonably good performance, which can both be improved or deteriorated, depending on the inclusion of extra side-streams. All investigated parameters were expressed as a percentage of the reference diet that was also included in the same trial. Calculation of the relative larval yield is shown in Equation (2) as an example, the other parameters (% BE; % LM weight after 14 or 28 days of rearing; % weight of feed and % costs) were calculated in a similar way.

(2) $$\%\;Larval\;yield=\frac{total\;biomass\;on\;diet\;}{average\;yield\;larvae\;on\;reference\;diet,\;measured\;in\;the\;same\;test\;set\;}\times 100.$$

All growth trials were performed eight times (number of trays per mixture). The data were expressed as averages with the standard deviation. The data were statistically processed by one-way analysis of variance (ANOVA) (*p* < 0.05) and a Tukey’s post hoc test by using IBM SPSS software. The Shapiro–Wilk test was used to control the data on a normally distribution (*p* < 0.05) and Levene’s test was used to judge the variance of the population (*p* < 0.05). When these terms were not met, a Kruskal–Wallis analysis was performed with a pairwise comparison, and significant values were adjusted by Bonferroni correction.

**Compliance with ethics requirements:** All applicable international, national, and/or institutional guidelines for handling of the animals were followed.

## 3. Results

### 3.1. Pure Side-Streams

The calculated percentage of larval yield, percentage larval weight, and percentage BE are reported in Table 3, along with the trial number and the relative amount of provided feed. Larvae that performed poorly processed the feed at a slower rate and were given, as such, less feed throughout the rearing experiment.

In the case of single side-streams with BG, larvae on the rice bran diet performed worse, which was deduced from lower feed provided and a lower percentage larval yield (40%). These larvae were also very small and therefore very difficult to separate from the substrate (before weighing and counting). Subsequently, no average larval weights after 14 days of rearing are available. Despite the relatively high BE, this diet was considered less ideal. The percentage larval yield for DDGS (79%) and corn gluten feed (84%) was higher but below the reference diet. However, the larval weight after 28 days for the larvae reared on DDGS was similar to that of the reference diet. 

When single side-streams were used in combination with grated carrots as a moisture source, the larvae performed worse. The percentage larval yield dropped below 40% for all side-streams except wheat middlings (74%). The fact that BG also provides nutrients besides moisture may explain the difference in performance. The growth performance on rice bran and corn gluten feed was inferior with low feed intake, low percentage larval yield, and low percentage BE. The larvae reared on rice bran were even too small to be counted and weighed at the end of the trial. Larvae reared with DDGS grew slightly better (higher percentage yield, percentage larval weight, and percentage BE), but a significantly improved performance was obtained with rapeseed meal. In general, the results indicate that wheat middlings and rapeseed meal provided sufficient nutrients for good larval rearing, also in the absence of BG. Concerning percentage larval yield, percentage larval weight, and percentage of BE, wheat middlings in combination with BG were proven to be the most successful diet.

### 3.2. Reference Diet

**Figure 1:**
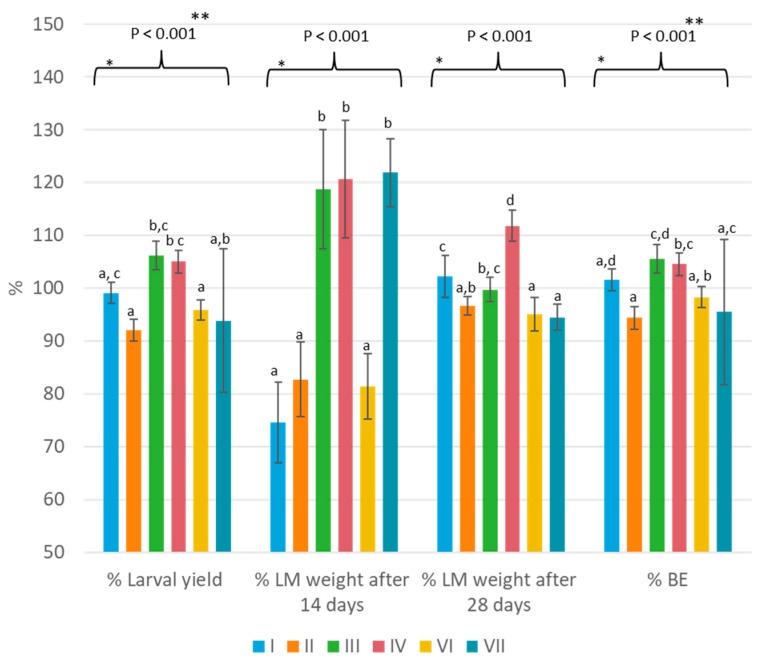
Parameters on growth performance (percentage larval yield, percentage LM weight, and percentage of BE) of the reference diet (wheat middlings supplemented with brewery grains (BG)) for six different trials (*n* = 8). The average of six different trials (I, II, III, IV, VI, VII) of the reference diet (each *n* = 8) was used as the denominator in Equation (2). * Within this data set, averages that are not labeled with the same letter are significantly different on the basis of the ANOVA-Tukey analyses. ** The data showed no equal variances of population, and the Kruskal–Wallis analysis was applied.

Data in Table 3 confirmed that the reference diet (diet F) resulted in the best larval production. Figure 1 provides more details on the larval performance parameters that were measured for diet F throughout all different trials and expressed as normalised values. On the basis of all six treatments with the reference diet, an actual average yield per tray of 992.96 ± 55.45 g was calculated, the actual average LM weight at 14 days was 2.38 ± 0.53 mg, and the actual average LM weight at 28 days was 20.01 ± 1.31 mg. Significant variations (*p* < 0.05) were notable for all four parameters. The percentage larval yield (*p* < 0.001) varied over the six trials between 92% and 106%. Trials II and VI resulted in significantly lower percentage yields compared to trials III and IV. The *p*-value for percentage larval weight at day 14 and day 28 were smaller than 0.001. In respect to percentage larval weight, after 14 days of rearing, trials III, IV, and VII had a significantly higher percentage (119%–122% versus 75%–83%), whereas after 28 days the values of all trials were situated between 95%–102%, except for trial IV (112%). The percentage of BE varied between 94% and 106%.

### 3.3. Mixed Side-Streams

The impact of different inclusions of a second side-stream in the reference diet (wheat middlings + BG) was evaluated by comparing the growth performance parameters to the reference diet (diet F). The data that was collected in different experiments over a time frame of six months are summarized in Table A1 (Appendix A). The data were normalized to the average of the reference diet that was included in the same trial. 

**Figure 2:**
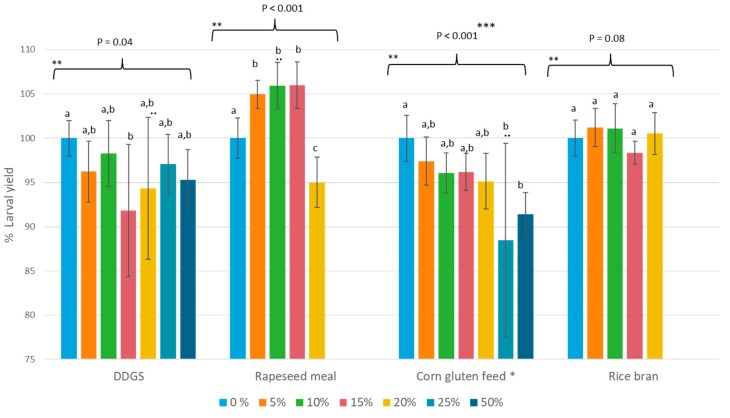
Percentage larval yield of the mixed diets grouped per side-stream. The bars indicate the percentage of wheat middlings that were replaced by the side-stream, where 0% refers to the reference diet (*n* = 8). * Reference diet was based on a fivefold repeat; ** within this data set, averages that are not labeled with the same letter are significantly different on the basis of the ANOVA-Tukey analyses. The average of the reference diet that was included in the same trial was used as the denominator in Equation (2). ** These data had a *p*-value of 0.03 for the Shapiro–Wilk test; *** these data showed no equal variances of population, and the Kruskal–Wallis analysis was applied.

Figure 2 illustrates the percentage larval yield for all mixed diets, grouped according to the type of the second side-stream. On the basis of the ANOVA assay, significant differences (*p* < 0.05) were detected between the inclusions of all side-streams except for rice bran. Replacement of wheat middlings by rice bran, up to 20%, did not significantly impact the percentage larval yield (*p*-value = 0.08). The same observation was made for lower inclusion percentages (0% until 15%) of corn gluten feed, whereas for a 25% or 50% replacement of the wheat middlings by corn gluten feed, a negative effect was observed. Partial replacement of wheat middlings by DDGS led to percentage larval yield ranging between 92% and 98%. A significant lower value was obtained with the 15% inclusion in comparison to the reference diet. All other inclusions had no significant impact. Rapeseed meal (tested up to an inclusion of 20%), on the other hand, resulted in a clear improvement on the percentage larval yield with 5% to 15% replacement of wheat middlings by rapeseed meal (105%–106%). A replacement of 20%, however, led to a significant decrease of percentage larval yield compared to the reference (0% inclusion). 

Because the amount of feed given during the trails was similar over all the diets, the results and conclusions based on the percentage of BE values were similar to the ones formulated for percentage larval yield.

**Figure 3:**
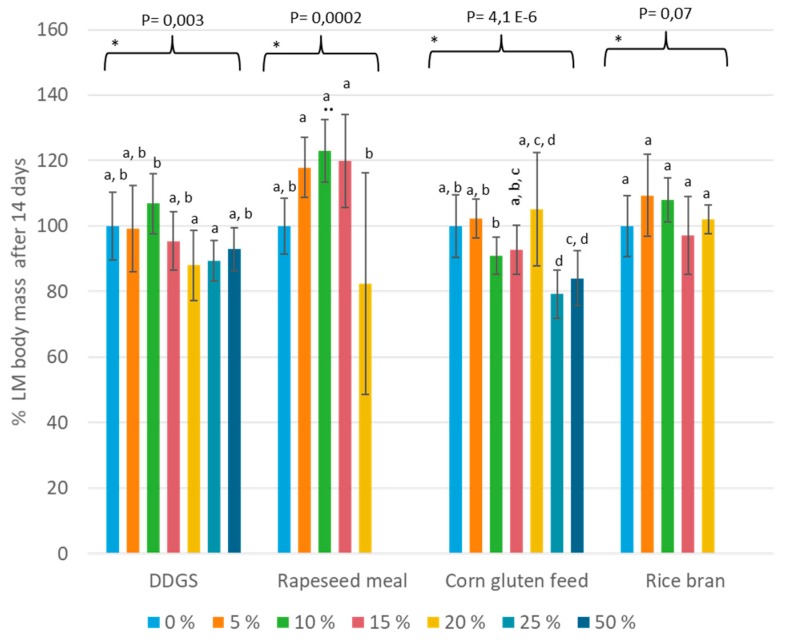
Percentage larval weight after 14 days of rearing for the mixed diets. The bars indicate the percentage of wheat middlings that was replaced by the side-stream, where 0% refers to the reference diet (*n* = 8). * Within this data set, averages that are not labeled with the same letter are significantly different on the basis of the ANOVA-Tukey analyses. The average of the reference diet that was included in the same trial was used as the denominator in Equation (2). This data had a *p*-value of 0.03 for the Shapiro–Wilk test.

The data related to percentage larval weight after 14 days of rearing are summarized in Figure 3. Again, inclusion of rice bran had no significant impact on the larval weight after 14 days of rearing (*p* > 0.05). The trials with DDGS revealed that a 10% replacement had a slightly but significantly higher larval weight at day 14 compared to a 20%–25% inclusion, but none of the mixtures were significantly different from the reference. Larvae reared with a 20% inclusion of rapeseed meal had a significantly lower larval weight compared to the other mixture, but again, no significant differences compared to the reference were calculated. For the trials that incorporated corn gluten feed, no diet was able to significantly increase the larval weight compared to the reference diet. Diets containing corn gluten feed had either no significant effect (up to 20%) or a negative effect (25% and 50%). 

**Figure 4:**
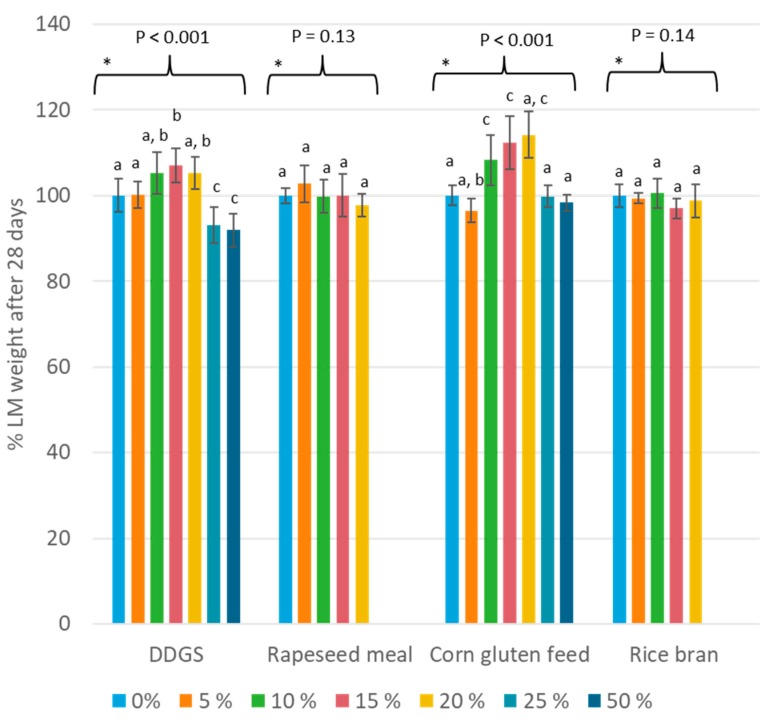
Larval weight after 28 days of rearing for the mixed diets. The bars indicate the percentage of wheat middlings that was replaced by the side-stream, where 0% refers to the reference diet (*n* = 8). * Within this data set, averages that are not labeled with the same letter are significantly different based on the ANOVA-Tukey analyses. The average of the reference diet that was included in the same trial was used as the denominator in Equation (2).

At the end of the rearing, the results and conclusions based on percentage larval weight (day 28) were different for some aspects (see Figure 4). Again, no significant changes in larval weight were detected when rice bran was incorporated (*p* = 0.14), but a similar conclusion was also true for rapeseed meal (*p* = 0.13) at day 28. A high percentage of DDGS resulted in a significant decreased larval weight, whereas at day 14 only a trend was observed. On the other hand, smaller incorporations of DDGS (15%) or corn gluten feed (10%–15%) slightly increased the larval weight above 100%, pointing towards a slightly but significantly positive trend. 

To evaluate the overall impact of the different diet inclusions, all growth parameters were summarised in Table A1, and data that were found to be significantly different from the reference diet (0% inclusion) based on the ANOVA-Tukey analyses were marked.

## 4. Discussion

The impact of side-stream inclusions (DDGS, rapeseed meal, corn gluten feed, and rice bran) in a reference diet (wheat middlings + BG) on the rearing of LM was evaluated by monitoring different growth parameters, these being larval yield, BE, and weight per larvae after 14 days and 28 days of rearing. It must be kept in mind that the diets were designed for the purpose of investigating the impact of side-streams, and that these are not standard insect rearing diets. The reference diet was included as a control in each of the trials that were performed over a period of several months. A variation in the growth parameters was observed for the reference diet, despite the fact that the trials were performed with first instar larvae of the same strain from a colony that was stable for more than 30 years. Only a different generation was used. These findings suggest that natural variations in the quality of the larvae may occur. This was also observed by Wang et al. (2017), who stated that natural variations among individuals and batches can be significant as they found a large range in protein and lipid content of black soldier fly larvae originating from the same company in three studies [17]. Rho and Lee (2016) also reported a variation in microbial load within rearing batches of the yellow mealworm, despite similar rearing protocols and intrinsic parameters [18]. In addition, variations in the side-streams may have also had an impact. The wheat middlings and BG used for these experiments were purchased in bulk, but not all trials were performed with meal from the same batch and thus a potential impact of seasonality, different storage times (mainly for BG), and the heterogenicity of the side-streams was inevitable [19], which could have influenced the rearing results. By normalising the data of the test sets to the control, a comparison between trials was made possible.

These normalised growth parameters (percentage larval yield, percentage of BE, percentage larval weight after 14 days and 28 days) were determined for each test condition. For most conditions, the percentage of BE and percentage larval yield were similar. However, for instance, for DDGS and rapeseed meal in Table 3, the percentage of BE values were similar to the percentage of BE of the reference diet, yet the percentage larval yield was significantly less. This underlines that the percentage of BE was not a suitable monitoring parameter for evaluating the efficiency of the growth of the larvae without considering other growth-related aspects such as larval yield and larval weight. Van Broekhoven et al. (2015) reported the same BE for two LM rearing batches reared on a different diet but the development time for the larvae to reach the pupal stage was significantly different. In addition, two rearing batches with a significantly different BE did provide larvae with the same larval weight [3]. Because BE in the current study was calculated on the basis of the provided amount of feed, underestimation was easily obtained, for example, when a significant amount of feed was not eaten by the larvae or when a restricted amount of feed inhibited optimal growth [4,20]. Depending on the rearing conditions chosen by the rearing facility, either an equal amount of feed is given for all trials (which might result in over- or underfeeding) [8] or feed is provided depending on the larval growth (which results in different feed gifts over all trials) [3]. The latter was chosen in this study. However, for diets containing BG (except for rice bran), the amount of feed provided was rather similar.

Considering all growth parameters measured (Table A1), the results that were obtained with DDGS inclusions in the diets did not reveal a clear impact pattern. A negative impact on larval yield was observed for 15% and 100% inclusion diets, whereas no significant impact was observed for the other inclusion percentages, and the larvae weight at the end was increased or similar for these inclusions. This can point to possible larval death (explaining the lower larval yield). Zhang et al. (2019) also found a 53% decreased larval weight when the YM was reared on a single beverage distiller’s grain (spirit distillers’ grains) compared to a wheat bran diet [21]. Nevertheless, a trend was seen towards decreasing growth parameters when the inclusion rate of DDGS was increased, pointing towards a reduced digestibility of specific compounds. 

In respect to rapeseed meal, LM growth data with 100% inclusion (with carrots, Table 3) already indicated that rapeseed meal provided a more balanced nutrient profile or the larvae were able to digest the nutrient better compared to DDGS, corn gluten feed, or rice bran. The increased larval weight after 14 days suggested that the nutrients in rapeseed meal may be beneficial at low doses for especially young larvae. All growth parameters indicated that, for the mixed diets (Table A1, an inclusion of 5% to 15% positively influenced the LM breeding process. In contrast, for the 20% inclusion diet with rapeseed, a negative impact was clearly obtained. This observation may be explained by glucosinolates, an anti-nutritional factor, present in rapeseed meal. Pracros et al. (1992) reported negative effects of glucosinolates, on the growth performance of the yellow mealworm. Different rapeseed meals with variating concentrations of the compound were tested, and only for one diet (concentration of glucosinolate of 26 µmol/100g dry diet) a decrease in larval performance was noticed due to metabolic problems. The larvae were able to survive but had a delay in weight gain. As the concentration of glucosinolates is different depending on the rapeseed species, no direct comparison to the current study is possible [22]. However, the data acquired in the current study suggest that this threshold was reached with a 20% inclusion of the rapeseed meal used in the current study. 

Corn gluten feed, as a single ingredient (with carrots as a moisture agent) was not found suitable for growing LM larvae. In mixed diets, lower inclusion percentages (5% to 20%) gave similar results compared to the reference diet, and Mancini et al. (2019) also found an improvement in YM larval weight when two worse-performing, single diets (cookie and bread remains) were combined [12]. Next, as was observed for rapeseed meal and DGGS, inclusion of higher percentages of corn gluten (25% and 50%) resulted in a statistically significant negative impact. Potentially, corn gluten also contains a compound that negatively impacts the growth.

With respect to rice bran, incorporation up to 20% had no significant effect on the larval rearing process. However, rice bran as a singular ingredient was shown not to be able to support good larval growth. When assuming that a 100% rice bran diet lacks the necessary nutrients, a 20% replacement of wheat middlings by rice bran would decrease the nutrients in the diet. Because the larval yield and weight remained the same, it could be presumed that nutrients were not limiting in 80% wheat middlings and that 20% of wheat middlings were inessential. Alternatively, this 20% of wheat middlings may have provided another function (non-nutritional) to the larvae. For example, it may have influenced the density of the growth medium and replacing it by rice bran did not change the settings. In any case, this observation offers the potential for replacing part of the nutrient-delivering ingredient by a cheaper component.

**Figure 5:**
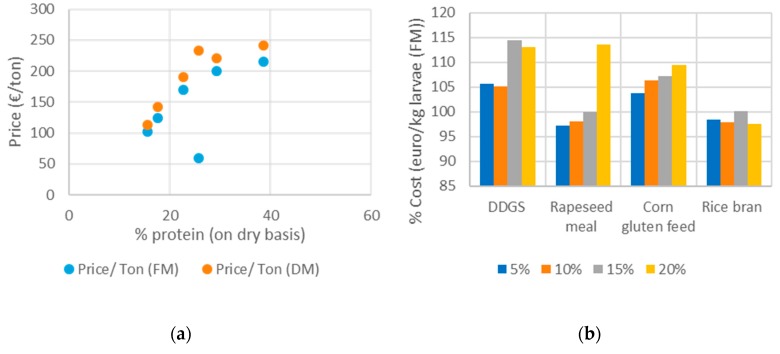
(**a**) Correlation between relation of protein content of side-streams and their price (in euros) on product basis (fresh matter, FM) and dry matter (DM) basis. (**b**) Effect on the cost price of side-stream inclusions in the reference diet compared to the cost price of the reference diet (100% cost).

To select the most optimal diet among the tested side-stream mixtures, not only larval growth performance but also economic aspects needed to be considered. Although raw material prices (see Table 1) fluctuate over time, the relative price difference may remain valid over a longer time period. Figure 5a shows that prices of the investigated side-streams were positively correlated with the protein content, especially when expressed on a dry matter basis. Considering the cost price and performance of the diets, the reference diet (wheat middlings and BG) was considered a relatively good fundamental ingredient for larval rearing. Figure 5b shows the percentage increase or decrease of the cost price of the feed compared to the reference diet in relation to the larval yield (calculated on the basis of Equation (2)). The use of DDGS and corn gluten feed would lead to an increase in production cost, whereas inclusion of rice bran, a much cheaper side-stream compared to wheat middlings, could lead to a lower production cost. Despite the relatively high price of rapeseed meal, an inclusion of 5% and 10% rapeseed meal would be cost yield-wise beneficial as a higher larval yield was obtained. Other aspects that influence the suitability of side-streams as an insect feed ingredient, such as availability of the side-streams, a site-specific and potentially seasonally dependent factor, are aspects to be considered more in detail.

## 5. Conclusions

The inclusion of side-streams in insect diets was investigated, envisioning a more economically favorable rearing. The obtained results prove that the lesser mealworm (LM) was able to survive on all side-streams tested in this study. However, the growth performance varied among side-streams. A reference diet of brewery grains and wheat middlings provided good results. Replacement of 5% to 15% of wheat middlings in this diet by rice bran or rapeseed meal was found possible without negatively effecting larval growth or production costs. Even reduced production costs were predicted. The results indicate that agri-food side-streams do have potential for insect diets. This offers opportunities for a wider search for cheaper and underspent side-streams to avoid competition with other feed applications and improve the economics of insect rearing.

## Figures and Tables

**Table 1:** Side-streams from the agri-food sector considered in this study. DM: dry matter.

	Cost Price (€/ton) ^1^	DM (%) ^2^	Protein Content on Dry Base (%) ^2^
**Corn DDGS**	200	90.3	29.3
**Rice Bran**	102	89.7	15.5
**Rapeseed meal**	215	88.9	38.7
**Corn gluten feed**	170	89.5	22.7
**Wheat middlings**	124	87.4	17.6

^1^ According to the supplier in the summer of 2017. ^2^ According to CVB (Centraal Veevoederbureau) table (2018) [15].

**Table 2:** Overview and inclusion percentages of the side-streams (expressed as fresh matter grams of side-stream per 100 g mixture) in the insect diets tested in this study.

Diet	Wheat Middlings	Rice Bran	Rapeseed Meal	Corn Gluten Feed	DDGS	Moisture Source ^1^	Trial ^2^
A	100					carrot	VIII
B		100				carrot	VIII
C			100			carrot	V
D				100		carrot	V
E					100	carrot	V
F	100					BG	I, II, III, IV, VI, VII
G		100				BG	IV
H				100		BG	III
I					100	BG	I
J	95	5				BG	IV
K	90	10				BG	IV
L	85	15				BG	IV
M	80	20				BG	IV
N	95		5			BG	II
O	90		10			BG	II
P	85		15			BG	II
Q	80		20			BG	II
R	95			5		BG	VII
S	90			10		BG	VII
T	85			15		BG	VII
U	80			20		BG	VII
V	75			25		BG	III
W	50			50		BG	III
X	95				5	BG	VI
Y	90				10	BG	VI
Z	85				15	BG	VI
AA	80				20	BG	VI
AB	75				25	BG	I
AC	50				50	BG	I

^1^ BG: brewery grains. ^2^ The trial number indicates the tests that were carried out in the same period.

**Table 3:** Parameters on growth performance related to experiments performed on pure side-streams, expressed as relative values (%) compared to the reference diet F (*n* = 8).

	Wheat Middlings	Rice Bran	Rapeseed Meal	Corn Gluten Feed	DDGS
Moisture Source: Brewery Grains *					
Trial	I, II, III, IV, VI, VII	VI		III	I
Diet	Diet F	Diet I	nd	Diet H	Diet I
% Weight of feed	100.0 ± 0.7 ^a^	55.0 ± 0.0 ^b^	nd	100.0 ± 0.0 ^a^	100.0 ± 0.0 ^a^
% Larval yield	100.0 ± 5.6 ^a^	38.1 ± 1.8 ^c^	nd	73.4 ± 2.7 ^b^	79.7 ± 3.7 ^b^
% LM weight after 14 days	100.0 ± 22.4 ^a^	na	nd	77.3 ± 7.3 ^b^	90.5 ± 5.5 ^a^
% LM weight after 28 days	100.0 ± 6.5 ^a^	43.5 ± 3.0 ^d^	nd	85.9 ± 2.7 ^c^	91.3 ± 5.2 ^b^
% BE	100.0 ± 7.1 ^a^	89.1 ± 4.1 ^b^	nd	77.6 ± 2.7 ^c^	78.1 ± 3.6 ^c^
Moisture Source: Grated Carrots **					
Trial	VIII	VIII	V	V	V
Diet	Diet A	Diet B	Diet C	Diet D	Diet E
% Weight of feed	106.6 ± 0.0 ^a^	23.8 ± 0.0 ^e^	57.4 ± 0.0 ^b^	26.2 ± 0.0 ^d^	35.8 ± 0.0 ^c^
% Larval yield	73.9 ± 1.3 ^a^	4.1 ± 0.5 ^d^	39.7 ± 1.4 ^b^	4.6 ± 0.2 ^d^	20.0 ± 0.4 ^c^
% LM weight after 14 days	88.3 ± 4.7 ^a^	na	142.5 ± 21.2 ^b^	na	na
% LM weight after 28 days	82.3 ± 2.7 ^a^	na	64.6 ± 10.4 ^b^	11.4 ± 1.1 ^d^	23.1 ± 1.6 ^c^
% BE	113.0 ± 2.0 ^a^	32.2 ± 4.0 ^c^	102.9 ± 3.7 ^b^	34.9 ± 1.8 ^c^	102.6 ± 2.1 ^b^

LM = lesser mealworm, BE = bioconversion efficiency, na = not available, nd = not determined. Averages that are not labeled with the same letter are significantly different on the basis of the ANOVA-Tukey analyses. * The average of the reference diet that was tested in the same trial was used as the denominator in Equation (2). ** The average of the reference diet (diet F) of six different trials (I, II, III, IV, VI, VII; each *n* = 8) was used as the denominator in Equation (2).

## Appendix A

**Table A1:** Growth and performance of lesser mealworm (LM) on the selected side-streams with BG as a moisture agent. * (*n* = 5).

	0%	5%	10%	15%	20%	25%	50%	100%
	means	means	means	means	means	means	means	means
DDGS								
% larval yield	100.0 ± 2.0	96.2 ± 3.5	98.3 ± 3.7	**91.8 ± 7.5 (** **−** **)**	94.3 ± 7.9	97.1 ± 3.4	95.3 ± 2.3	**79.8 ± 3.7 (** **−** **)**
% BE	100.0 ± 2.0	96.1 ± 3.5	98.1 ± 3.7	**91.5 ± 7.4 (** **−** **)**	93.9 ± 7.9	96.6 ± 3.4	94.3 ± 2.3	**78.1 ± 3.6 (** **−** **)**
% LM weigh after 14 days	100.0 ± 10.2	99.2 ± 13.1	106.9 ± 9.2	95.5 ± 8.9	88.0 ± 10.7	89.4 ± 6.3	92.9 ± 6.6	90.5 ± 5.5
% LM weight after 28 days	100.0 ± 3.9	100.1 ± 3.0	105.3 ± 4.8	**107.0 ± 4.0 (+)**	105.2 ± 3.7	**93.1 ± 4.2 (** **−** **)**	**91.9 ± 3.9 (** **−** **)**	**91.3 ± 5.2**
Rapeseed meal								
% larval yield	100.0 ± 2.3	**104.9 ± 1.6 (+)**	**105.9 ± 2.6 (+)**	**105.9 ± 2.6 (+)**	**95.0 ± 2.8 (** **−** **)**			
% BE	100.0 ± 2.3	**104.9 ± 1.6 (+)**	**105.8 ± 2.6 (+)**	**105.8 ± 2.6 (+)**	**94.7 ± 2.8 (** **−** **)**			
% LM weigh after 14 days	100.0 ± 8.6	117.9 ± 9.1	123.0 ± 9.6	119.9 ± 14.2	82.4 ± 33.8			
% LM weight after 28 days	100.0 ± 1.8	102.7 ± 4.3	99.8 ± 3.8	100.1 ± 4.9	97.7 ± 2.7			
Corn gluten feed								
% larval yield *	100.0 ± 2.6	97.4 ± 2.7	96.1 ± 2.3	96.2 ± 2.1	95.1 ± 3.1	**92.0 ± 5.0 (** **−** **)**	**91.4 ± 2.5 (** **−** **)**	**79.4 ± 2.7 (** **−** **)**
% BE *	99.9 ± 2.7	97.3 ± 2.7	95.3 ± 2.3	95.9 ± 2.1	94.7 ± 3.1	**91.4 ± 5.0 (** **−** **)**	**90.3 ± 2.5 (** **−** **)**	**77.6 ± 2.7 (** **−** **)**
% LM weigh after 14 days	100.0 ± 9.5	102.3 ± 6.0	90.9 ± 5.7	92.8 ± 7.4	105.2 ± 17.4	**79.2 ± 7.4 (** **−** **)**	**84.1 ± 8.5 (** **−** **)**	**77.3 ± 7.3**
% LM weight after 28 days	100.0 ± 2.3	96.5 ± 2.8	**108.2 ± 5.8 (+)**	**112.4 ± 6.2 (+)**	114.2 ± 5.5	99.8 ± 2.5	98.3 ± 1.8	**85.9 ± 2.7**
Rice bran								
% larval yield	100.0 ± 2.0	101.2 ± 2.2	101.1 ± 2.8	98.4 ± 1.3	100.5 ± 2.4			**38.1 ± 1.8** **(−** **)**
% BE	100.1 ± 2.0	101.04 ± 2.1	100.8 ± 2.8	97.9 ± 1.3	99.9 ± 2.4			89.1 ± 4.1
% LM weigh after 14 days	100.0 ± 9.2	109.4 ± 12.6	108.0 ± 6.6	97.1 ± 12.0	102.0 ± 4.5			/
% LM weight after 28 days	100.0 ± 2.6	99.3 ± 1.2	100.5 ± 3.5	96.9 ± 2.4	98.7 ± 3.9			**43.5 ± 3.0**

The data were normalized to the average of the reference diet that was included in the same trial; numbers in bold refer to values that were found significantly different from the reference diet (0% inclusion) on the basis of ANOVA-Tukey analyses. Positive impacts are marked with (+), whereas negative effects are marked with (−).
